# The Effect of *MUC1* rs4072037 Functional Polymorphism on Cancer Susceptibility: Evidence from Published Studies

**DOI:** 10.1371/journal.pone.0095651

**Published:** 2014-04-22

**Authors:** Fujiao Duan, Chunhua Song, Liping Dai, Shuli Cui, Xiaoqin Zhang, Xia Zhao

**Affiliations:** 1 Department of Hospital Infection Management, Affiliated Tumor Hospital of Zhengzhou University, Henan Tumor Hospital, Zhengzhou, Henan, China; 2 Department of Epidemiology, College of Public Health, Zhengzhou University, Zhengzhou, Henan, China; 3 Henan Key Laboratory of Tumor Epidemiology, Zhengzhou, Henan, China; 4 College of Professional Study, Northeastern University, Boston, Massachusetts, United States of America; University of North Carolina School of Medicine, United States of America

## Abstract

Genome-wide association studies have identified a susceptibility variation *MUC1* rs4072037 for gastric cancer in Chinese population. Subsequent case-control studies have reported this association in other populations. However, the results remain controversial and ambiguous. The aim of this study is to provide a precise quantification for the association between *MUC1* rs4072037 variation and the risk of cancer. We performed pooled analysis of 10 case-control designed studies including 4,220 cases and 6,384 controls. Odds ratios (OR) and 95% confidence interval (95%CI) were calculated to assess strength of association in overall studies and in subgroup analysis stratified by ethnicity and cancer types. All statistical analyses were performed by Manager 5.0 and Stata 12.0 software. Overall, the *MUC1* rs4072037 polymorphism was associated with risk of cancer in all genetic models (G vs A: OR = 0.71, 95%CI: 0.63–0.80, *p*<0.01; GA vs AA: OR = 0.61, 95%CI:0.55–0.67, *p*<0.01; GG vs AA: OR = 0.58, 95%CI: 0.47–0.71, *p*<0.01; AG+AA vs GG: OR = 0.60, 95%CI: 0.55–0.60, *p*<0.01; GG vs AG+AA: OR = 0.70, 95%CI: 0.58–0.85, *p*<0.01). Further, subgroup analysis based on ethnicity suggested *MUC1* rs4072037 polymorphism had a subtly reduced cancer risk among Asian population, and stratified analysis by cancer types showed significantly decreased risk of gastric cancer in all genetic models. In conclusion, *MUC1* rs4072037 polymorphism may be used as potential biomarker for cancer susceptibility particularly for gastric cancer and for Asian population.

## Introduction

Cancer is the leading cause of death in economically developed countries and the second leading cause of death in developing countries. The global burden of cancer continues to increase largely because of the aging and growth of the world population alongside an increasing adoption of cancer-causing behaviors [1]. Based on the GLOBOCAN 2008 estimates, about 12.7 million cancer cases and 7.6 million cancer deaths are estimated to have occurred in 2008 [2]. Cancer arises due to the complex interplay of genetic and environmental risk factors, genetic predisposition has been considered to be a combined influence factor [3], the inheritance of the majority of cancers is polygenic [4].

In past decades, a large number of association studies were carried out in different cancers, but only few convincing low penetrance susceptible loci have been identified until now [5]. Recently, Genome-wide association studies (GWAS) play important roles in the identification of potential candidates for single nucleotide polymorphisms (SNPs). In 2010, Abnet et al. [6] conducted a GWAS on gastric cardia adenocarcinoma (GCA) and identified a statistically significant SNP of rs4072037 in mucin1 (*MUC1*) gene, which located at 1q22 is a synonymous SNP in the second exon of *MUC1*, pointing intricate role of *MUC1* in malignancy.

The *MUC1* gene, encodes cell-surface glycoprotein with a complex cytoplasmic domain thought to be involved in signal transduction [7], which is associated with attenuation of intracellular levels of reactive oxygen species (ROS), as well as epithelial cancers and with poor survival [8], [9]. However, it remains unknown whether this locus is associated with an increased risk of all cancer types. Because GCA is a digestive system disease, it may manifest through different molecular mechanisms. In addition, the GWAS was carried out in a Han Chinese population. Therefore, we have extensively reviewed the literature and performed a meta-analysis based on all eligible published case-control studies to evaluate the association between *MUC1* rs4072037 polymorphism and cancer susceptibility.

## Materials and Methods

### Identification and Eligibility of Relevant Studies

This meta-analysis was conducted by searching PubMed, Elsevier, Springer, and the Chinese Biomedical Literature database (CBM) for relevant published articles in English and Chinese (updated to December, 2013). Combination of the key words were as follows: ‘*MUC1* polymorphism’, ‘*MUC1* 586 A/G’, ‘rs4072037’ and ‘1q22 polymorphism cancer’. Eligible studies were examined carefully to determine whether they accorded with the inclusion criteria for the meta-analysis. The studies included must meet the following criteria: a) about the rs4072037 polymorphism and cancer risk, b) from a case-control designed study, c) sufficient published data for estimating an odds ratio (OR) with 95% confidence interval (CI), and d) genotype frequencies available.

The studies, in which the genotype of controls for a certain polymorphism was not meet the assumptions for Hardy-Weinberg equilibrium (HWE), were excluded from the analysis.

### Data Extraction

Two authors independently extracted data and entered them in a customized database. For conflicting evaluations, an agreement was reached after a discussion. For each eligible study, the following data were extracted: first author’s name, publication year, country of origin, ethnicity, pathological type, study design, detecting sample, genotype method, numbers of genotyped cases and controls and HWE of controls, respectively. Ethnic descents were categorized as Asian or Caucasian. Study design was stratified into hospital-based or population-based studies. If original genotype frequency data were not available in relevant studies, a request for additional data was sent to the corresponding author.

### Statistical Analysis

Statistical analyses were performed by Manager 5.0. The risks of cancer associated with rs4072037 polymorphism were calculated directly from the data given in the eligible studies. ORs and corresponding to 95%CI were used to assess the strength of association between rs4072037 polymorphism and cancer risk. The pooled ORs were performed for allelic comparison (G vs A), heterozygote comparison (GA vs AA), homozygote comparison (GG vs AA), dominant model (AA+GA vs GG), and recessive model (AA vs GA+GG), respectively. Furthermore, the analyses were stratified by ethnicity (Asian, and Caucasian), cancer type (if only one cancer type contained fewer than two individual studies it was combined into the ‘Other Cancers’ group) and source of controls.

We assessed the departure from the HWE for the control group in each study using Pearson’s goodness-of-fit *χ^2^* test with 1 degree of freedom.

Heterogeneity across studies was evaluated by Cochran’s Q test and *I^2^*, which represents the percentage of total variation across studies that is attributable to heterogeneity. Heterogeneity was considered statistically significant when *P*<0.05 or *I^2^*>50%, then the pooled OR estimate of each study was calculated by the random-effects model [10]. Otherwise, the fixed-effect model was used [11]. The significance of overall odds ratio (OR) was determined by the Z-test. If there were significant heterogeneity among included studies, the sources of heterogeneity would be explored using meta regression in Stata version 12.0 (http://www.stata.com).

To assess the stability of the results, sensitivity was carried out by omitting each study in turn to assess the quality and consistency of the results. Publication bias was assessed using funnel plots, Begg’s test (rank correlation test), and Egger’s test (weighted linear regression test) for funnel plot symmetry [12]. These analyses were performed using Stata version 12.0 software.

Statistical tests performed in the present study were considered significant whenever the corresponding null-hypothesis probability was *P*<0.05.

## Results

### Characteristics of the Studies

**Figure 1:**
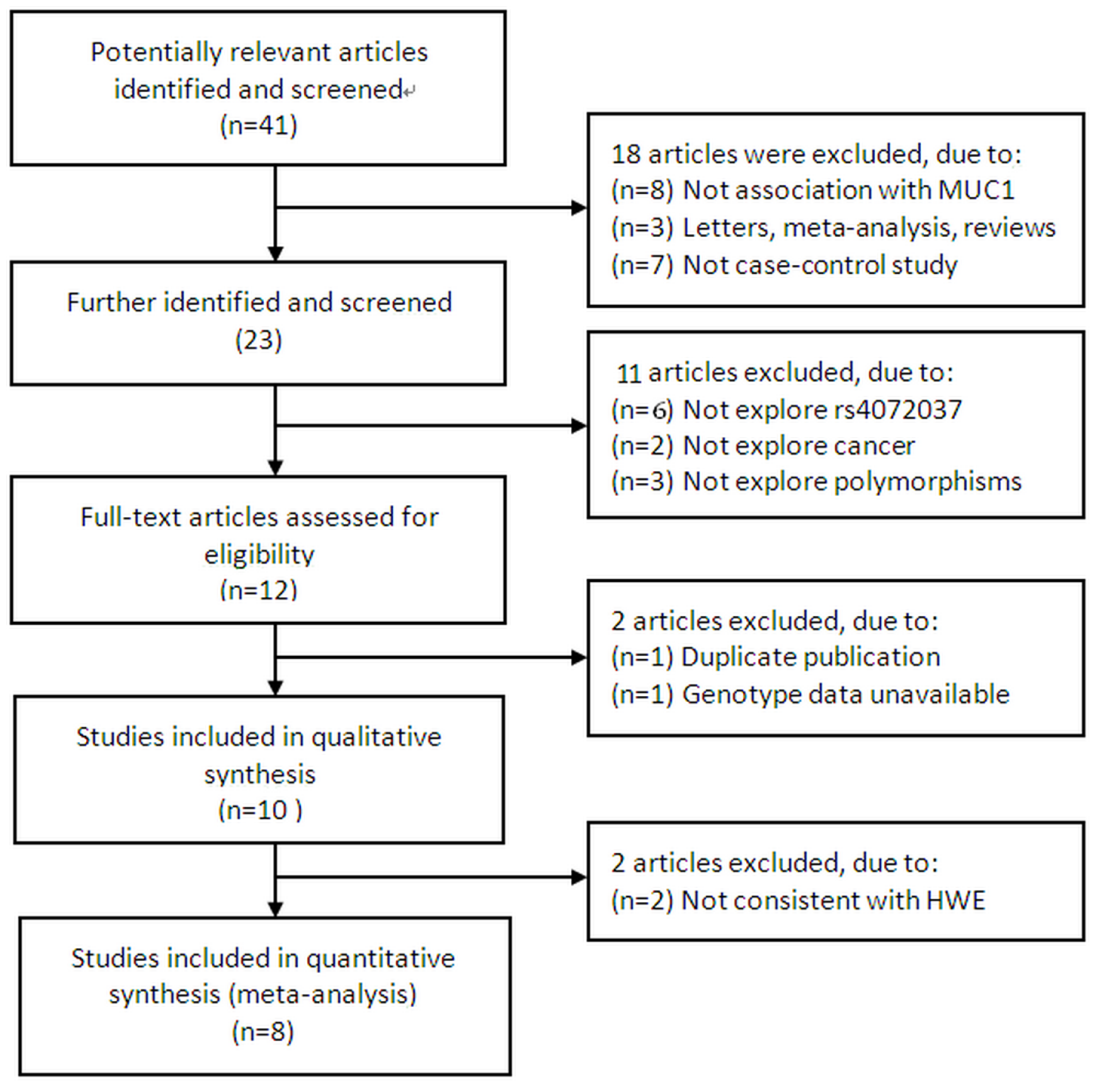
Flow chart of literature search and study selection.

Based on the search criteria and inclusion criteria, a total of 13 studies available from 10 articles for *MUC1* rs4072037 polymorphism were investigated[13]–[21], the study selection process is shown in Figure 1, as summarized in Table 1, in which 3 studies (two articles) [15], [21] genotype of controls for a certain polymorphism were not consistent with HWE, were excluded from the analysis. Hence, a total of 10 studies including 4,220 cases and 6,384 controls were used in the meta-analysis. All studies were case-control designed, including 3 cancer types, in which 8 studies on gastric cancer, and one each on colorectal cancer and breast cancer. There were 8 studies of Asian descendent and 2 of Caucasian descendent. A classic TaqMan assay was used in 4 out of 10 studies. 7 studies were randomly repeated a portion of samples as quality control while genotyping.

**Table 1:** Characteristics of literatures included in the meta-analysis.

Author[Ref]	Year	Study type	Country	Ethnicity	Cancer type	Source of control	Genotyping	Matching criteria	Case/Control	Quality control	*P*_HWE_
Li M [13]	2013	Case-control	China	Asian	Gastric Cancer	Population	TaqMan	Age; sex	335/334	Y	0.242
Li FX [14]	2012	Case-control	China	Asian	Colorectal cancer	Hospital	MassARRAY	NA	231/292	NA	0.434
Palmer AJ [15]	2012	Case-control	United States	Caucasian	Gastric Cancer	Population	TaqMan	Age; sex	311/207	NA	0.024
Palmer AJ [15]	2012	Case-control	United States	Caucasian	Esophageal cancer	Population	TaqMan	Age; sex	159/207	NA	0.024
Zhang H [16]	2011	Case-control	China	Asian	Gastric Cancer	Population	TaqMan	Age; sex; area	1658/1833	Y	0.335
Saeki N [17]	2011	Case-control	Japan	Asian	Gastric Cancer	Population	TaqMan	Age; sex	605/1264	Y	0.466
Saeki N [17]	2011	Case-control	Japan	Asian	Gastric Cancer	Population	TaqMan	Age; sex	303/1467	Y	0.110
Saeki N [17]	2011	Case-control	Korea	Asian	Gastric Cancer	Population	PCR-LDR	Age; sex	449/369	Y	0.391
Abnet CC [6]	2010	GWAS	China	Asian	Gastric Cancer	Population	Chip	Age; sex	2240/3302	Y	NA
Jia YB [18]	2010	Case-control	Poland	Caucasian	Gastric Cancer	Population	SNPlex	Age; sex	272/376	Y	0.483
Xu Q [19]	2009	Case-control	China	Asian	Gastric Cancer	Population	PCR-SSPs	Age; sex	138/241	NA	NA
Kruit A [20]	2008	Case-control	Netherlands	Caucasian	Breast cancer	Hospital	PCR-SSPs	NA	229/208	NA	0.243
Strawbridge RJ [21]	2008	Case-control	Sweden	Caucasian	Prostate Cancer	Population	Beckman	NA	67/130	Y	0.010

NA: not available; HWE: Hardy-Weinberg equilibrium.

### Quantitative Synthesis

**Figure 2:**
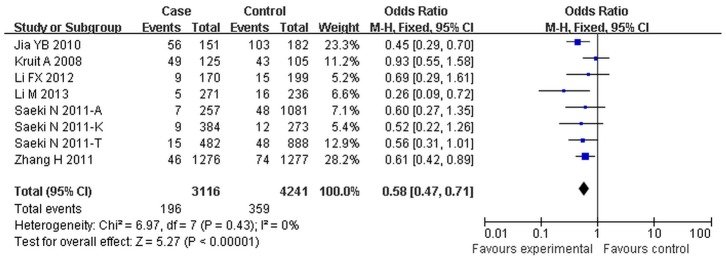
Forest plot of cancer risk associated with *MUC1* rs4072037 for homozygous genetic model (GG vs AA). The squares and horizontal lines correspond to the study-specific OR and 95%CI. The area of the squares reflects the study specific weight. The diamond represents the pooled OR and 95%CI.

The pooled analyses and the heterogeneity test are shown in Table 2 and Figure 2. A significantly decreased cancer risk was found in all genetic models, including the allelic genetic model (G vs A: OR = 0.71, 95%CI: 0.63–0.80, *p*<0.01), heterozygous genetic model (GA vs AA: OR = 0.61, 95%CI:0.55–0.67, *p*<0.01), homozygous genetic model (GG vs AA: OR = 0.58, 95%CI: 0.47–0.71, *p*<0.01), the dominant model (AG+AA vs GG: OR = 0.60, 95%CI: 0.55–0.60, *p*<0.01), and the recessive model (GG vs AG+AA: OR = 0.70, 95%CI: 0.58–0.85, *p*<0.01).

**Table 2:** Main results of pooled ORs in the meta-analysis.

Comparisons	Cases	Controls	Heterogeneity test			Summary OR *(95% CI)*	Hypothesis test		Studies
	n/N	n/N	*Q*	*P*	*I^2^*(%)		*Z*	*P*	
G vs A	1907/12372	3669/18888	21.70	0.01	63	0.71(0.63,0.80)	5.78	<0.01	9
AG vs AA	955/3875	1901/5783	8.24	0.31	15	0.61(0.55,0.67)	10.04	<0.01	8
GG vs AA	196/3116	359/4241	6.97	0.43	0	0.58(0.47,0.71)	5.27	<0.01	8
AG+GG vs AA	1173/4220	2321/6384	8.93	0.35	10	0.60(0.55,0.65)	11.03	<0.01	9
GG vs AG+AA	196/4082	359/6143	5.77	0.57	0	0.70(0.58,0.85)	3.12	<0.01	8

Further stratification analysis by ethnicity, the results showed that *MUC1* rs4072037 polymorphism was significantly linked to cancer risk (table 3). Overall, individuals carrying G allelic had a subtly reduced cancer risk among Asian population (G vs A: OR = 0.69, 95%CI: 0.61–0.79, *p*<0.01; GA vs AA: OR = 0.61, 95%CI:0.55–0.67, *p*<0.01; GG vs AA: OR = 0.56, 95%CI: 0.43–0.73, *p*<0.01; AG+AA vs GG: OR = 0.60, 95%CI: 0.54–0.66, *p*<0.01; GG vs AG+AA: OR = 0.64, 95%CI: 0.49–0.82, *p*<0.01). In Caucasian population, *MUC1* rs4072037 polymorphism was significantly associated with an decreased cancer risk in all genetic models except for homozygous genetic model and recessive model (G vs A: OR = 0.76, 95%CI: 0.64–0.90, *p*<0.01; GA vs AA: OR = 0.61, 95%CI:0.46–0.80, *p*<0.01; AG+AA vs GG: OR = 0.58, 95%CI: 0.44–0.75, *p*<0.01).

**Table 3:** Stratified analyses of the *MUC1* rs4072037 polymorphism on cancer risk.

Comparisons	Heterogeneity test			Summary OR *(95%CI)*	Hypothesis test		Studies
	*Q*	*P*	*I^2^*(%)		*Z*	*P*	
Ethnic							
*Asian*							
G vs A	17.26	0.01	65	0.69(0.61,0.79)	5.44	<0.01	7
AG vs AA	6.73	0.24	26	0.61(0.55,0.67)	9.42	<0.01	6
GG vs AA	2.66	0.75	0	0.56(0.43,0.73)	4.40	<0.01	6
AG+GG vs AA	7.15	0.31	16	0.60(0.54,0.66)	10.26	<0.01	6
GG vs AG+AA	2.40	0.79	0	0.64(0.49,0.82)	3.48	<0.01	6
*Caucasian*							
G vs A	3.89	0.05	74	0.76(0.64,0.90)	3.14	<0.01	2
AG vs AA	1.52	0.22	34	0.61(0.46,0.80)	3.47	<0.01	2
GG vs AA	4.20	0.04	76	0.64(0.32,1.29)	1.25	0.21	2
AG+GG vs AA	1.70	0.19	41	0.58(0.44,0.75)	4.25	<0.01	2
GG vs AG+AA	1.92	0.17	48	0.81(0.61,1.08)	1.45	0.15	2
Cancer subtypes							
*Gastric cancer*							
G vs A	17.32	0.01	65	0.68(0.60,0.77)	5.93	<0.01	7
AG vs AA	6.14	0.29	19	0.59(0.53,0.66)	9.93	<0.01	6
GG vs AA	3.04	0.69	0	0.52(0.44,0.66)	5.50	<0.01	6
AG+GG vs AA	6.83	0.34	12	0.58(0.53,0.64)	10.86	<0.01	6
GG vs AG+AA	2.40	0.79	0	0.65(0.52,0.80)	3.95	<0.01	6
*Other cancers*							
G vs A	0.87	0.35	0	0.86(0.70,1.06)	1.42	0.16	2
AG vs AA	0.00	1.00	0	0.74(0.56,1.00)	1.99	0.05	2
GG vs AA	0.35	0.55	0	0.85(0.54,1.33)	0.70	0.49	2
AG+GG vs AA	0.02	0.88	0	0.72(0.55,0.95)	2.35	0.02	2
GG vs AG+AA	0.46	0.50	0	0.97(0.65,1.45)	0.17	0.87	2

**Figure 3:**
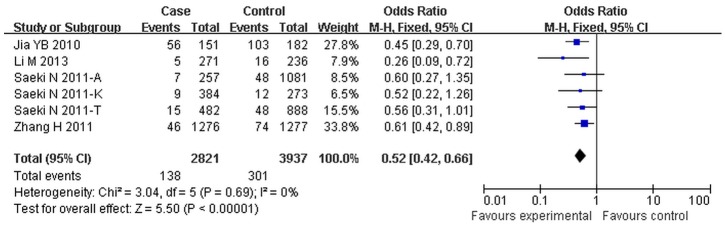
Forest plot of cancer risk associated with *MUC1* rs4072037 for the homozygous genetic model (GG vs AA) stratified by cancer types. The squares and horizontal lines correspond to the study-specific OR and 95%CI. The area of the squares reflects the study specific weight. The diamond represents the pooled OR and 95%CI.

Subgroup analysis was also stratified by cancer types, in different types of cancer, *MUC1* rs4072037 polymorphism was significantly associated with an decreased risk of gastric cancer in all genetic models (G vs A: OR = 0.68, 95%CI: 0.60–0.77, *p*<0.01; GA vs AA: OR = 0.59, 95%CI:0.53–0.66, *p*<0.01; GG vs AA: OR = 0.52, 95%CI: 0.44–0.66, *p*<0.01; AG+AA vs GG: OR = 0.58, 95%CI: 0.53–0.64, *p*<0.01; GG vs AG+AA: OR = 0.65, 95%CI: 0.52–0.80, *p*<0.01). No significant associations were found in the “others” group (colorectal cancer and breast cancer) except the dominant model (AG+AA vs GG: OR = 0.72, 95%CI: 0.55–0.95, *p*<0.01) (Table 3, Figure 3).

When studies were segregated based on source of control, *MUC1* rs4072037 polymorphism was found to be associated with decreased cancer risk, the results were equal to subgroup analysis of cancer subtypes (Table 3).

### Test of Heterogeneity

When evaluating the association between the *MUC1* rs4072037 polymorphism and the susceptibility to cancer, we found that there was significant heterogeneity for the allelic comparison (G vs A: *P*_heterogeneity_ = 0.01, *I^2^* = 63%). Thus, we assessed the source of heterogeneity for the allelic genetic model comparison, meta regression in Stata 12.0 was used to assess by cancer type, matched controls (yes or not), source of control (population or hospital), assay, sample size (500 as the boundary) and quality control (with or not). It was detected that the systemic results were not affected by these characteristics (data not shown).

### Sensitivity Analysis

Sensitivity analyses were performed to assess the influence of each individual study on the pooled ORs by the systematic omission of the individual study from the analyses. The corresponding pooled ORs were not materially altered, indicating that our results were statistically robust (data not shown).

### Publication Bias

**Figure 4:**
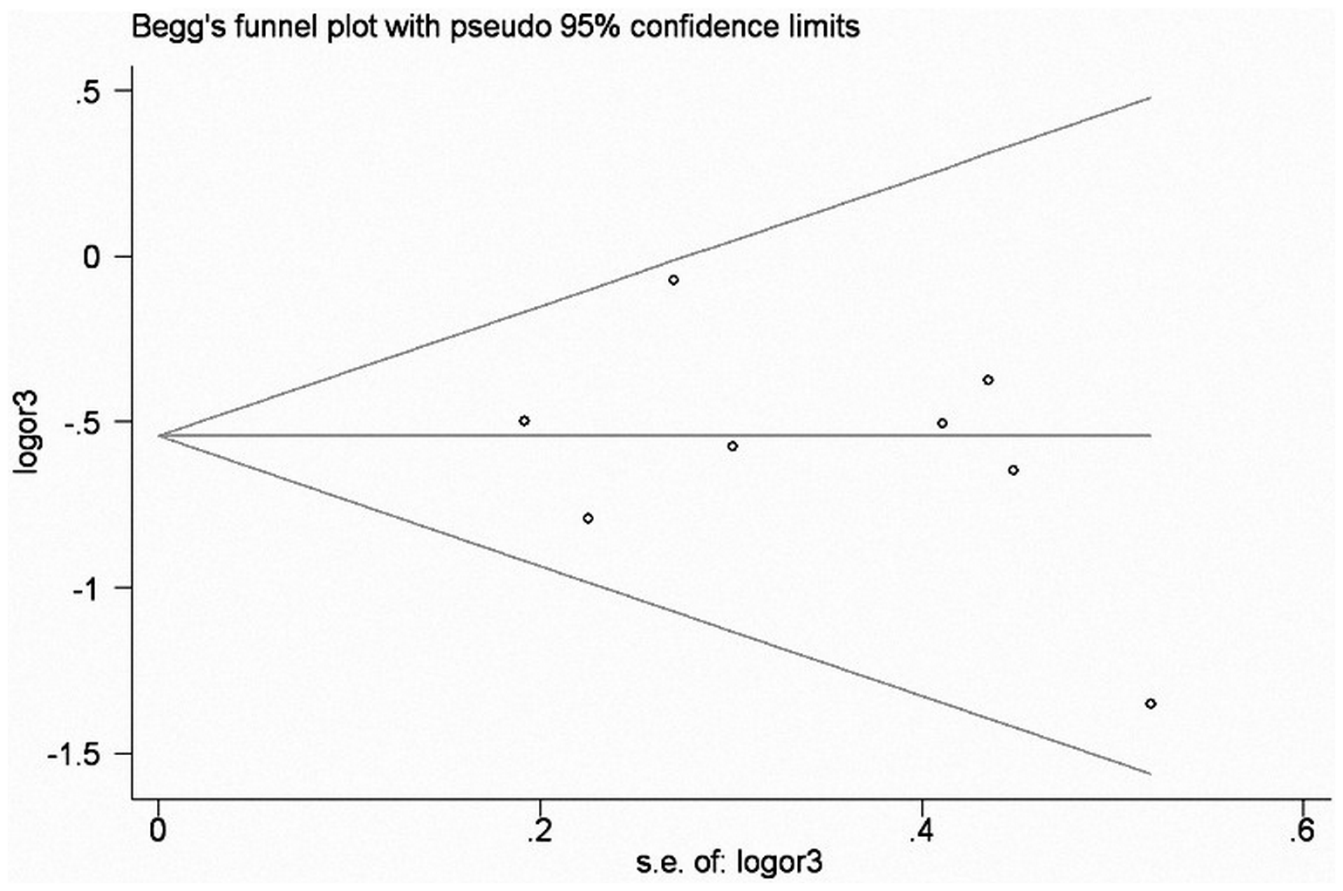
Funnel plot of *MUC1* rs4072037 polymorphism and cancer risk for homozygous genetic model (GG vs AA).

Begg’s funnel plot and Egger’s linear regression test were performed to assess the publication biases of included studies. The shapes of the funnel plots did not reveal any evidence of obvious asymmetry under the dominant model (Figure 4). Then, the Egger’s test was used to provide statistical evidence for funnel plot symmetry (Table 4).

**Table 4:** Publication bias of *MUC1* rs4072037 for Egger’s test.

comparisons	*t*	*p*	95% CI
G vs A	−0.30	0.77	−3.96∼3.07
AG vs AA	−1.13	0.30	−3.67∼1.35
GG vs AA	−0.57	0.59	−3.42∼2.13
AG+GG vs AA	−0.99	0.36	−4.05∼1.71
AA+AG vs GG	−1.05	0.34	−3.34∼1.34

## Discussion

*MUC1* is an extracellular protein anchored to the epithelial surface and engage in morphogenetic signal transduction [22]. Rs4072037 was observed to disrupt the physiological functions of *MUC1* [19], which might due to alternative splicing of the 5′-region of exon 2 controlled by rs4072037 and ultimately result in failure to the physiological protection of gastric mucosa [23].

GWAS was referred to as an unbiased and fairly comprehensive approach to explore the genetic variation related to cancer. Abnet et al. [6] reported the strong association of the new and novel low-penetrance susceptibility locus rs4072037 with the decreased risk of gastric cancer in the Chinese population by a large-scale GWAS. Replications of the association identified in GWAS by other independent studies were performed, but the study populations were not all Chinese, and the results were conflicting and inconclusive because of various reasons, such as different ethnicities, resident areas, sample size, and environmental factors. Meta-analysis, as an important statistical method has more statistical power than a single study, which can quantitatively combine analyses from different studies. To provide a more comprehensive analysis on the association, we producted this meta-analysis based on ten case-control designed studies with 10,568 participants.

In the present study, we firstly analyzed the association of *MUC1* rs4072037 of cancer in the overall cancer types. The results of our meta-analysis indicated that the G allele of *MUC1* rs4072037 variation was significantly associated with decreased risk of cancer. In addition, subgroup analyses revealed that *MUC1* rs4072037 G allele was associated with a decreased risk for progress of gastric cancer,especially in Asians. It indicated that a possible ethnic difference in the genetic background. Moreover, similar results have been obtained by Zheng et al [24]. The results suggested that *MUC1* rs4072037 polymorphism might be involved in gastric carcinogenesis and tumor differentiation among Asian populations. The results may be important for interpretation of genetic variance for susceptibility to gastric cancer and facilitate to be used as diagnostic markers in cancer, and are under investigation as therapeutic targets for cancer.

Although meta-analysis is robust, our meta-analysis had some limitations common to these types of studies. Firstly, because only published studies were retrieved in the meta-analysis, publication bias might be possible, even though the statistical test did not show it. Secondly, our meta-analysis did not evaluate any potential gene-gene interaction and gene-environment interaction due to lack of relevant published data. Thirdly, although perfect searching strategy was used to identify eligible studies for current meta-analysis, it was still possible that a few studies meeting inclusion criteria were not included.

In summary, this meta-analysis suggested that the *MUC1* rs4072037 polymorphism was significantly associated with decreased risk of cancer, especially for gastric cancer and Asian population. However, further well-designed studies in large cohort of different ethnic origins and cancer types are needed before the application of *MUC1* rs4072037 polymorphism as cancer biomarker in clinical settings.

## Supporting Information

Checklist S1 **PRISMA Checklist.**
(DOC)
